# Sp2 promotes invasion and metastasis of hepatocellular carcinoma by targeting TRIB3 protein

**DOI:** 10.1002/cam4.2977

**Published:** 2020-03-11

**Authors:** Yue Zhu, Jie Cui, Jiatao Liu, Wei Hua, Wei Wei, Guoping Sun

**Affiliations:** ^1^ Department of Oncology The First Affiliated Hospital of Anhui Medical University Hefei China; ^2^ Department of Pharmacy The First Affiliated Hospital of Anhui Medical University Hefei China; ^3^ Institute of Clinical Pharmacology Anhui Medical University Hefei China

**Keywords:** endoplasmic reticulum stress, hepatocellular carcinoma, prognosis, Sp2, TRIB3

## Abstract

**Objective:**

To explore the biological function and molecular mechanism of Sp2 in hepatocellular carcinoma (HCC).

**Methods:**

Tissue microarray immunohistochemistry and western blot were used to study the expression of Sp2 in hepatocellular tissue and adjacent non‐neoplastic tissues (ANT). In HCC cell lines, the role of Sp2 was determined by in vitro experiments such as CCK8, clone formation test, Transwell assay, wound‐healing assay, and flow cytometry apoptotic analysis, and its possible mechanism was analyzed.

**Results:**

Compared with ANT, Sp2 expression in HCC tissues was significantly up‐regulated, which was strongly associated with stage of tumor and poor prognosis of patients. TCGA database were further confirmed these results. Besides, functional studies had shown that Sp2 knockdown not only leads to a decrease in cell proliferation and an increase in cell apoptosis but also inhibits the cells' abilities of migration and invasion. Sp2 silencing could inhibit the expression of TRIB3 protein and down‐regulate the endoplasmic reticulum stress (ERS) level of HCC.

**Conclusion:**

Sp2 may play a part in promoting cancer by regulating TRIB3 protein, which may be a factor of prognostic and a potential new therapeutic target for HCC.

## Graphical abstract

We found that Sp2 expression was significantly up‐regulated in HCC tissues, which was closely related to tumor staging and poor prognosis of patients. Functional studies had shown that Sp2 knockdown not only leads to a decrease in cell proliferation and an increase in cell apoptosis, but also inhibits migration and invasion of hepatoma cells. Sp2 may play a role in promoting cancer by regulating TRIB3 protein, which may be a prognostic factor and a potential new therapeutic target for HCC.

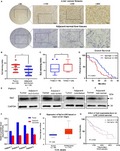

## 1 BACKGROUND

Primary hepatocellular carcinoma (HCC) is one of the most common malignant tumors in the clinic. Its incidence rate ranks sixth among all malignant tumors and its mortality rate ranks second.^1^, ^2^ Therefore, it is of vital importance to deeply explore the molecular basis behind the invasion and metastasis of HCC. Previous data show that endoplasmic reticulum stress (ERS) stress in human body is activated continuously, which has a close relationship to the occurrence and development of cancer,^3^, ^4^ which can not only repair damaged proteins or degrade misfolded proteins,^5^ protect tumor cells from body damage,^6^ but also regulate the growth and apoptosis of tumor cells, promote tumor invasion and metastasis^7^, ^8^ through various ways, and mediate chemotherapy resistance.^4^ In the endoplasmic reticulum stress environment, TRIB3 is up‐regulated as a sensor of cell stress, which is related to poor prognosis and involved in the progress of tumors.^9^

Transcription factor Sp2 is a member of the SP protein family, which is involved in the regulation of cell proliferation, differentiation, apoptosis and other functions.^10^ Recent studies have shown that Sp2 may overexpress and play a crucial part in many kinds of cancers.^11^, ^12^ However, the role of Sp2 in HCC has not been studied. The aim of this research was to clarify the relationship between Sp2 expression in HCC and clinicopathological features by detecting the expression of Sp2, TRIB3, and ERS marker protein GRP78 in HCC and adjacent non‐neoplastic tissues (ANT), further study the biological function of Sp2 in hepatoma cells by small interfering RNA (siRNA) transfection technology so as to clarify its influence on proliferation, apoptosis, and invasion of HCC cells, as well as explore its possible molecular mechanism.

## 2 METHODS

### 2.1 Clinical samples

The clinical samples of 95 pairs of patients with HCC and corresponding ANT from the First Affiliated Hospital of Anhui Medical University from 2004 to 2010 were collected. The specific data of the studied patients including the age, gender, size of tumor, histological grade, Lymphatic metastasis, and clinical stage were recorded for further study. Tumor clinical stage was classified on the basis of TNM stage of the International Union Against Cancer (UICC). In addition, we also obtained matched four pairs HCC/ ANT liver tissue samples from surgical patients to carry out the western blot. Our scientific research program follows the relevant moral standards officially issued in the Declaration of Helsinki, and is reviewed and finally approved by the ethical standards committee of our hospital. All patients involved in the study have signed the informed consent in written form.

### 2.2 Immunohistochemistry (IHC)

Ninety‐five pairs of tissue microarrays of HCC/ANT samples were constructed. Rabbit polyclonal anti‐human Sp2 antibody (1:100, Biorbyt), TRIB3 antibody (1:400, Bioss), GRP78 antibody (1:500, Arigo) was immunohistochemically detected by two‐step method. Positive signal intensity scores were as follows: 1 point is negative (not stained); 2 point is weak (light yellow); 3 point is medium (yellow brown); 4 point is strong (brown). The positive degree was scored according to the actual proportion of positive cells: 0 point corresponds to <5%; 1 point corresponds to 5%‐25%; 2 points correspond to 26%‐50%; 3 points correspond to 51%‐75%; 4 points correspond to more than 75%. The staining index (SI) was taken as the total score obtain a range of 0‐16. In addition, the median SI was equal to 8 as the cutoff value. For the samples with SI <8 and SI ≥8, they correspond to the samples of low and high expression group, respectively.

### 2.3 Cell culture

The human HepG2, Huh7, and Hep3B cell line used in this study were all from the Chinese Academy of Sciences and were identified by Wuhan Procell Co., Ltd. The HepG2 cells and Hep3B cells were cultured in DMEM medium (Gibco) containing streptomycin (100 μg/mL, Gibco), penicillin (100 U/mL, Gibco), and 10% fetal bovine serum (HyClone, FBS). The Hub7 cells were cultured in RPMI‐1640 medium (Hyclone) containing the same substances as mentioned above. Cells were cultured in 37℃ incubator containing 5% carbon dioxide.

### 2.4 Small interfering RNA (siRNA) and small hairpin RNA (shRNA) transfection

HCC cells were plated and grown in six‐well plates before transfection at approximately 50% confluence. The transfection process completely in accordance with the instructions of the manufacturer. Lipofectamine TM 2000 (Invitrogen) was used to finish this process. Small interfering RNA for the Sp2 (si‐Sp2) were obtained from the GenePharma Co.,Ltd. Small hairpin RNA plasmids for the TRIB3 gene (shTRIB3) were obtained from the Genechem Co.,Ltd. The target sequences for SP2 were 5′‐ GGAGCAUCAUCAGCCUGAATT −3′ (sense) and 5′‐ UUCAGGCUGAUGAUGCUCCTT −3′ (antisense). The primer sequences for TRIB3 were 5′‐ TGGATGACAACTTAGATACCG −3′ (sense) and 5′‐ CGGTATCTAAGTTGTCATCCA −3′ (antisense). A non‐silencing siRNA （si‐NC） sequence (target sequence 5′‐ UUCUCCGAACGUGUCACGUTT −3′(sense) and 5′‐ ACGUGACACGUUCGGAGAATT −3′ (antisense)) and a non‐silencing shRNA (shCtrl) sequence (target sequence 5′‐ TTCTCCGAACGTGTCACGT −3′) sequence were used as the negative control.

### 2.5 Cell viability analysis

Cells were seeded into 96‐well plates with a seeding volume of 100 μL (density 3 × 104 cells/mL) per well, incubation temperature was 37℃, and the CO_2_ concentration was 5%. After 12 hours, 24 hours, 48 hours, and 72 hours of culture. Add CCK‐8 detection reagent (BestBio) (10 μL/well), and measure the absorbance at 450 nm.

### 2.6 Annexin V‐FITC/PI apoptosis detection

Forty‐eight hours after transfection, use the Annexin V‐FITC/PI staining method to accurately analyze apoptosis in each group. Cells were collected in the reaction tube and prepared into single cell suspension. PBS which has been precooled in advance was used to wash the above samples. After adding 5 μL of annexin V‐FITC and PI (eBiosciences) and avoided light for 15 minutes, apoptosis was detected using the Beckman CytoFLEX LX flow cytometry system (Beckman Coulter).

### 2.7 Transwell assay

A 24‐well Transwell chamber (Corning) with gelatin‐coated polycarbonate membrane filter and Matrigel were used. Cells were collected and suspended in the medium without serum to a cell density of 5 × 104/mL. Volume of 100 μL was added to the upper cavity of the transfer well. The lower rooms added 600 μL of medium contained 20% serum (HyClone, FBS). Twenty‐four hours later, the cells were dyed with crystal violet (0.1%) for 15 minutes, and then washed with PBS twice. The cells were observed and counted using ImageJ. Cells were observed by light microscope, and then randomly selected different areas (5 for each) to count.

### 2.8 Wound‐healing assay

The cells were collected efficiently and then inoculated into a six‐well plate(5 × 105/well). Cells were incubated and cultured to the level of 90%. The HCC cells were scraped using 200 μL tips and incubated in the serum‐free medium. The typical images have been collected under microscope at 0 hour, 12 hours, and 24 hours after scratch creation.

### 2.9 Western blot assay

Total protein was extracted and then quantified. SDS‐PAGE was used to separate protein and transfer them to PVDF membranes (Millipore). Incubate in skim milk (5%) at room temperature for 2 hours. And then, primary antibody was incubated at 4°C, including anti‐Sp2 (1:100 dilution, Biorbyt), anti‐TRIB3 (1:1000 dilution, Abclonal), anti‐GRP78 (1:500 dilution, Arigo), anti‐ATF6 (1:500 dilution, Bioworld), anti‐PERK (1:1000 dilution, Bioworld). Incubate with the corresponding secondary antibody (with dilution of 1:10 000) for a total of 120 minutes at 37°C. Finally, the protein band signal was observed with an enhanced chemiluminescence reagent (ThermoFisher).

### 2.10 Statistical methods

In this study, the mean ± standard deviation is used to represent the experimental data effectively. Statistical tests for data analysis included Log‐rank test, Fisher's exact test, chi‐squared test, Kaplan‐Meier analysis and paired/unpaired *t* test. Images were plotted using Graph Prism 8.0 software. SPSS software (version 20.0) was selected for statistical analysis of data. *P* < .05 means statistically significant.

## 3 RESULTS

### 3.1 Sp2 Overexpression is closely related to progression and adverse prognosis of HCC

**Figure 1:**
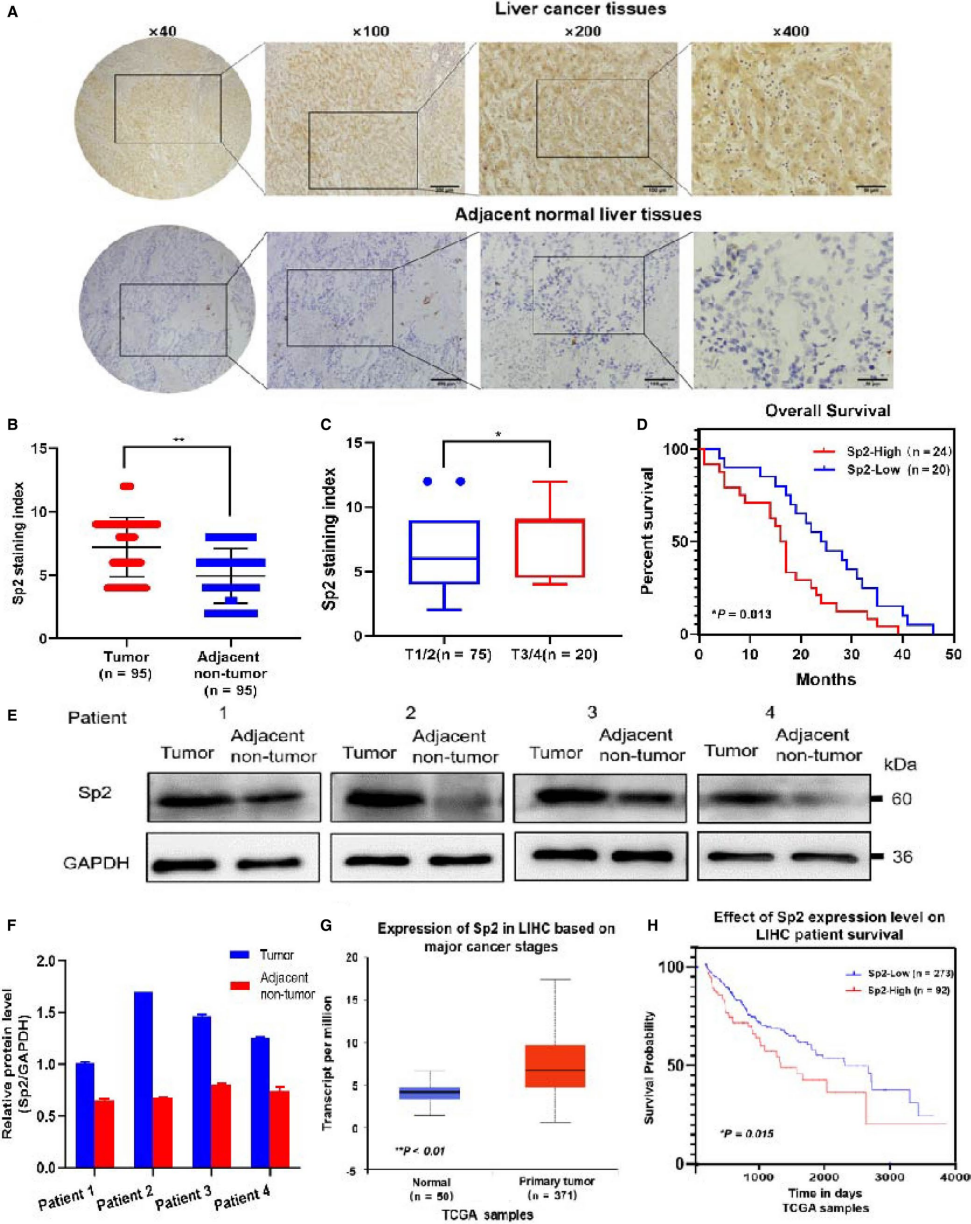
Sp2 overexpression is closely related to hepatocellular carcinoma (HCC) progression and poor prognosis. A, The expression of Sp2 in the microarray of HCC. The HCC tissue is brown, strong nuclear staining, and surrounding non‐cancerous liver tissue clearly showed that Sp2 staining was weak positive or negative under the microscopic. B, The staining index (SI) of Sp2 in tumor tissues of HCC patients was higher than ANT liver tissues. C, Sp2 protein levels in HCC tissues were related to TNM stage. D, Kaplan‐Meier survival curve with low and high Sp2 expression levels of HCC patients (*P* = .013, n = 44). E, F, Western blot was used to confirmed SP2 expression in fresh matched (4 pairs) adjacent non tumor tissues and corresponding HCC tissues. G, H, TCGA data proved that Sp2 was the low expression and high expression in normal tissues and liver hepatocellular carcinoma (LIHC) tissues, respectively (G). Kaplan‐Meier survival curve of LIHC measured at different Sp2 expression levels (corresponding to high and low expression) (n = 365; *P* = .015) (H). *P < .05 **P < .01

Evaluate the express of Sp2 in 95 cases of HCC/ANT samples by IHC method (Figure 1A). Sp2 was highly expressed in 54.7% (52/95) HCC tissues, but prominently lower in ANT liver tissues (Figure 1B). After detailed clinicopathological analysis, it was found that the expression of Sp2 was positively correlated with tumor size (*P* = .041), lymph node metastasis (*P* = .032), clinical stage (*P* = .011), but did not relate to the histological grade, gender and age of patients (Figure 1C; Table 1). Survival analysis showed low Sp2 expression means better survival benefit than high expression (Figure 1D). Western blot was used to detect the expression of Sp2 in 4 pairs of fresh HCC and its surrounding tissues, so as to verify the Sp2 expression pattern in HCC. The results showed that there is a significant overexpression of SP2 in HCC tissues compared with the surrounding corresponding tissues (Figure 1E,F). Analysis of paired HCC/ ANT liver tissue data set in The Cancer Genome Atlas (TCGA) showed that Sp2 expression in tumor tissues was obviously higher than normal liver tissues in patients with HCC (Figure 1G). TCGA data also provide a Kaplan‐Meier survival analysis showed there was a significant positive correlation between the high expression of Sp2 and the relatively short overall survival (Figure 1H). Based on these results, we can conclude that the overexpression of SP2 may be associated with the occurrence and evolution of HCC. In addition, for HCC, it may also be a new prognostic and diagnostic indicator.

**Table 1:** Sp2 expression in 95 hepatocellular carcinoma samples

Variable	n	Sp2 expression		*χ2* value	*P* value
		high	low		
Age(years)					
<60	56	32	24	0.319	.572
≥60	39	20	19		
Gender					
Male	69	37	32	0.126	.722
Female	26	15	11		
Tumor size(cm)					
≤5	40	17	23	4.176	.041
>5	55	35	20		
Histological grade					
Low‐intermediate	62	35	27	0.354	.552
High	33	17	16		
Lymph node metastasis					
Negative	80	40	40	4.589	.032
Positive	15	12	3		
TNM stage					
I‐II	75	36	39	6.526	.011
III‐IV	20	16	4		

### 3.2 Knockdown of Sp2 inhibits the growth of HCC cells

**Figure 2:**
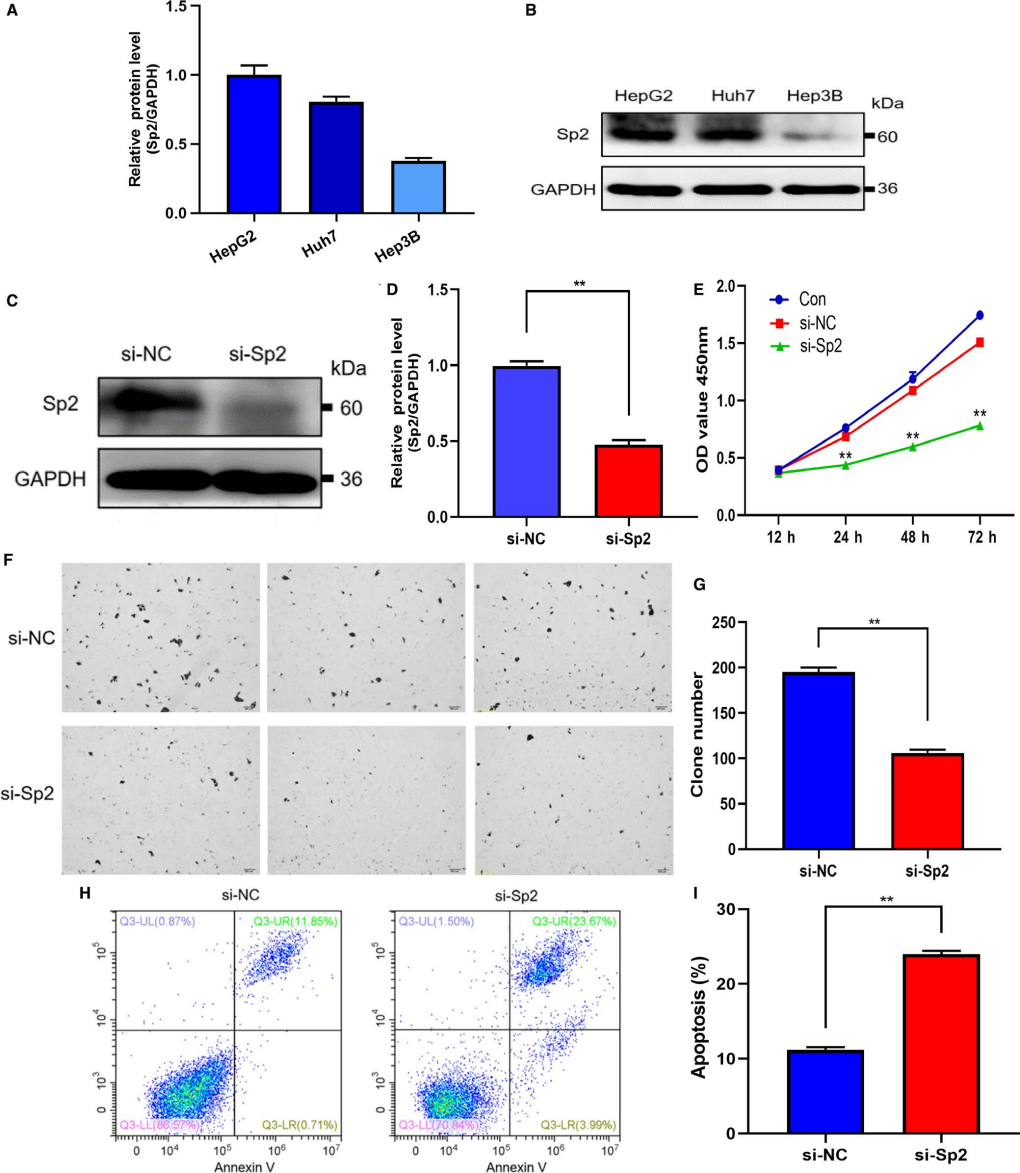
Effect of Sp2 knockdown on cell proliferation, colony formation, and apoptosis in HepG2 cells. A, B, Western blot was used to evaluate the Sp2 protein level of three common HCC cell lines. C, D, In HepG2 cells, siRNA interference fragments (targeted Sp2) were transiently transfected (C). Western blot was used to evaluate the silencing effect (D). E, Cell Viability images over three days in control HepG2 cells, si‐NC and si‐Sp2 HepG2 cells. F, G, After Sp2 knockdown treatment, use the colony formation to evaluate the growth of HepG2 cells effectively. H, I, The apoptosis rate was evaluated in si‐NC and si‐Sp2 HepG2 cells by flow cytometry. *P < .05 **P < .01

**Figure 3:**
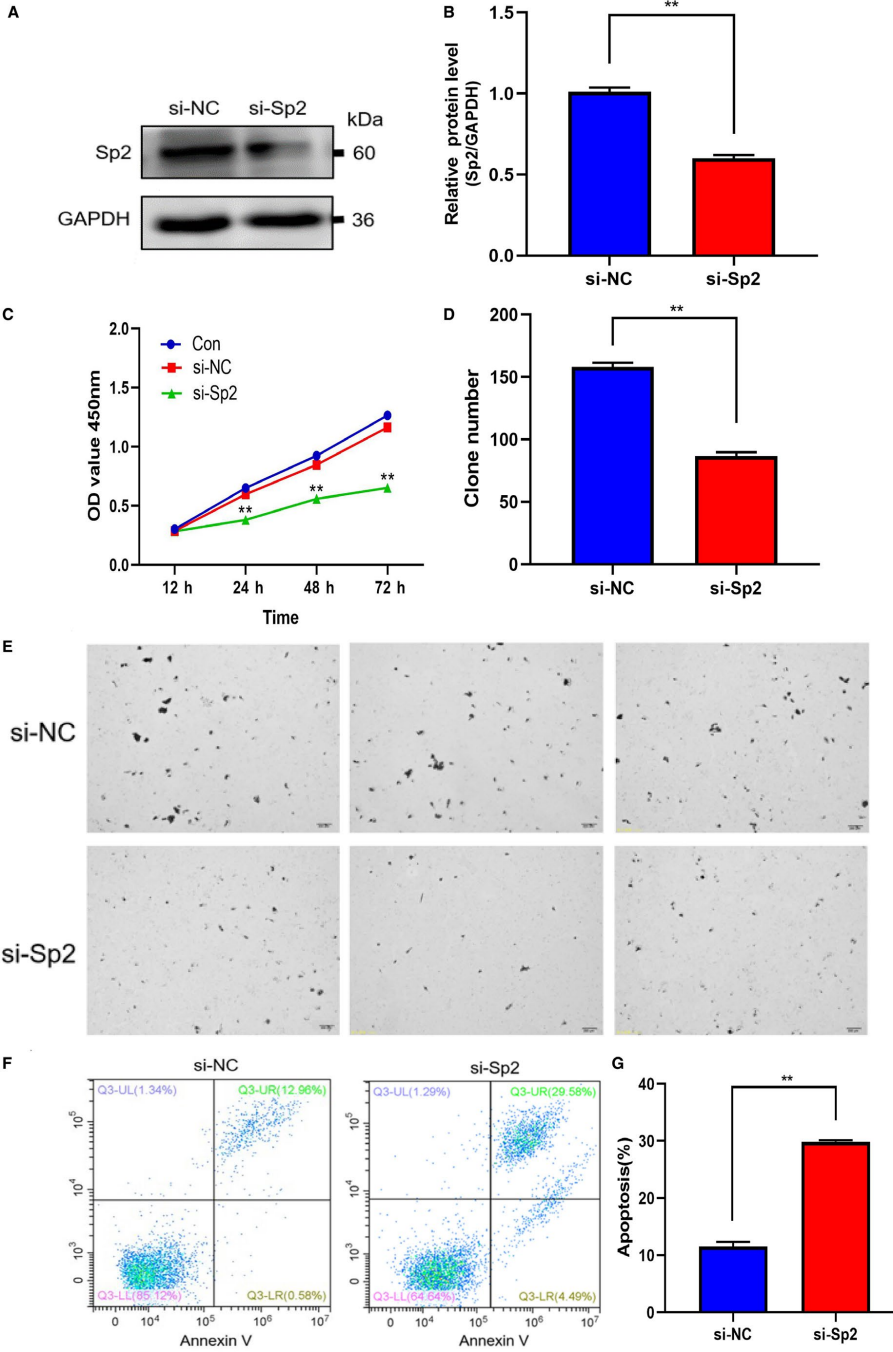
Effect of Sp2 knockdown on cell proliferation, colony formation and apoptosis in Huh7 cells. A,B, The Sp2‐targeted siRNA interfering fragments were transiently transfected into Huh7 cells, and the silencing efficacy has been evaluated via western blot. C, Cell Viability images over three days in control Huh7 cells, si‐NC and si‐Sp2 Huh7 cells. D,E, Assessment the growth of Huh7 cells, which have been treated with SP2 knockdown, has been performed through colony‐forming techniques. F,G, The apoptosis rate of si‐NC Huh7 cells and si‐Sp2 Huh7 cells. **P < .01

Western blot was selected to accurately detect the expression of Sp2 in HCC cell lines (Hep3B, Huh7, HepG2), so as to evaluate its biological function in vitro. Finally, HepG2 cell lines with high expression level were selected for subsequent studies (Figure 2A,B). The Sp2‐targeted siRNA interfering fragments were transiently transfected into HepG2 cells, Sp2 expression was significantly inhibited at the protein level (Figure 2C,D), which means that the construction of the model has been completed. To evaluate the cell proliferation, we used the colony‐forming assay and CCK8. Flow cytometry was used to explore the apoptosis of Sp2 silenced HepG2 cells. It was found that Sp2 deletion inhibited cell proliferation (Figure 2E) and colony formation (Figure 2F,G). In addition, inhibition of Sp2 also significantly increased apoptosis (Figure 2H,I). For purpose of further verify whether there was a certain degree of cell specificity in the biological function of Sp2, we additionally detected the hepatoma Huh7 cell line in vitro. Similarly, in Sp2‐silenced Huh7 cells (Figure 3A,B), cell proliferation was decreased (Figure 3C,D), colony formation was inhibited (Figure 3E), and apoptosis was increased (Figure 3F,G). These results indicated that Sp2 knockdown has the ability to inhibit the growth of cells and significantly promote cell apoptotic in the in vitro hepatoma cell line.

### 3.3 Knockdown of Sp2 inhibits invasion and migration of HCC cells in vitro

**Figure 4:**
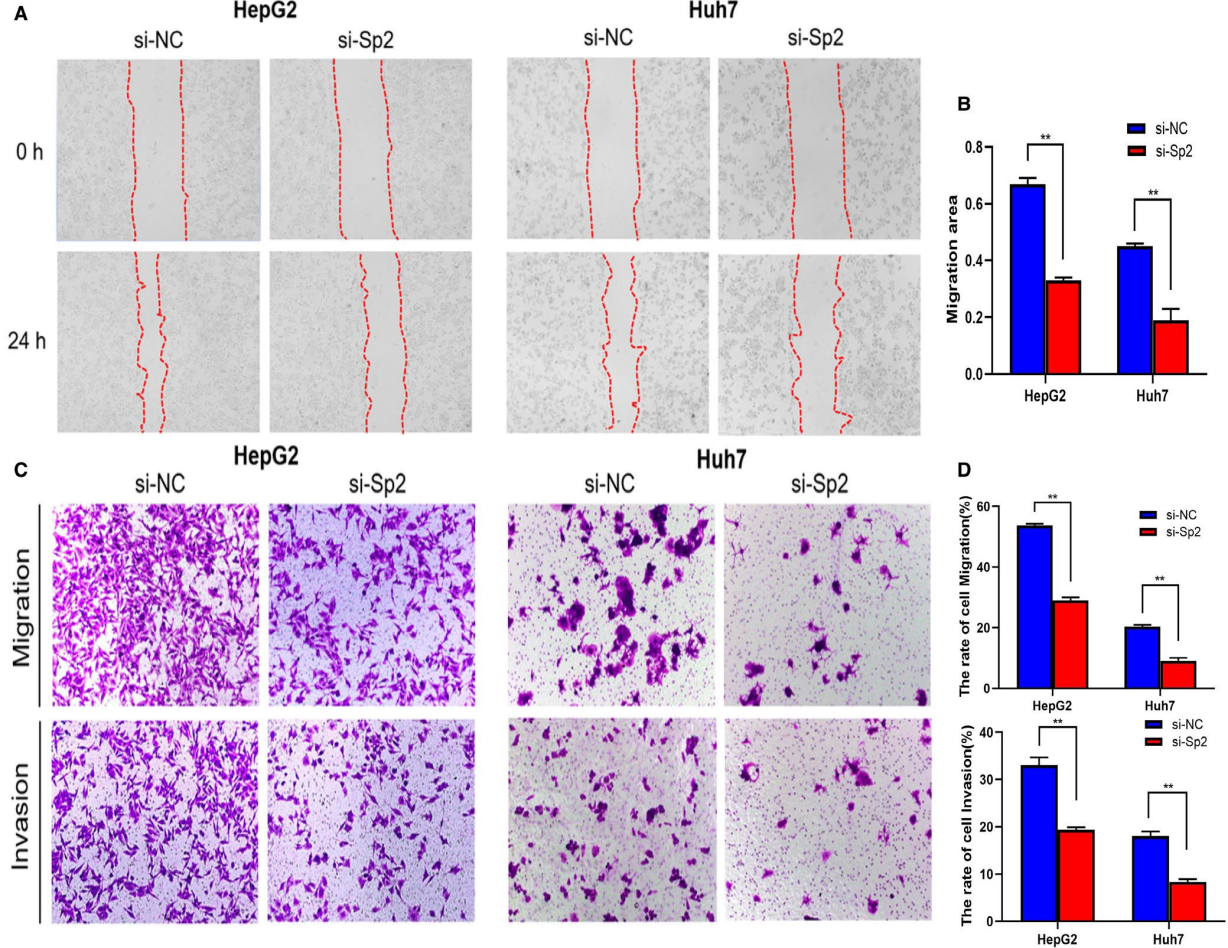
Effect of Sp2 knockdown on migration and invasion in HCC cells. A, The cell migration potential was evaluated by the wound healing test strictly referring to the time point (si‐NC and si‐SP2 cells) (original magnification, ×100). B, The migration areas of Sp2 silenced cells and cells from the control group were determined scientifically. C, Measure the cell migration and invasive ability by transwell assay (original magnification, ×100). D, Quantification of migration and invasion of si‐NC and si‐Sp2 cells. **P < .01

Our clinical results showed Sp2 overexpression was positively correlated not only with tumor stage but also with lymph node metastasis. Accordingly, it can be considered that SP2 should have a certain impact on tumor migration and invasion. Wound‐Healing test indicated that the healing rate of HepG2 and Huh7 cells after Sp2 knockout was significantly slowed down after scratch creation (Figure 4A,B). At the same time, the Transwell assay showed a significant decrease in cell migration and invasive ability after Sp2 knockout in both cell lines (Figure 4C,D). It also indicated that Sp2 was an important factor to promote invasion and migration of HCC cells in vitro.

### 3.4 Knockdown of Sp2 down‐regulates ERS

**Figure 5:**
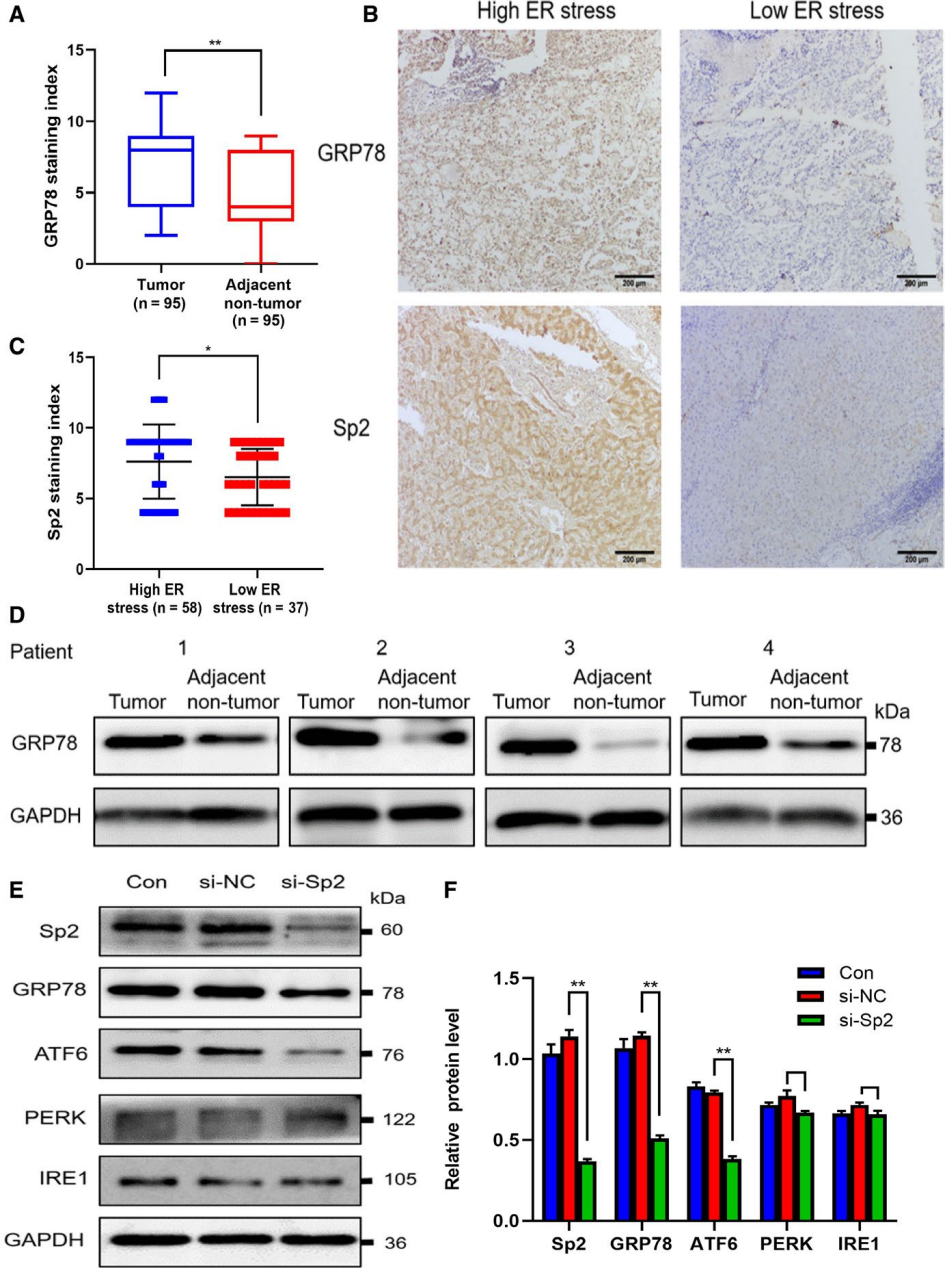
GRP78 expression in HCC and its relationship with Sp2. A, Compared with the surrounding normal tissues, the expression of GRP78 had a higher staining index (SI). B,C, Sp2 had a higher expression trend in the high ERS HCC tissues. D, The expression of GRP78 in primary HCC tissues and matched normal tissues (4 pairs in total) from the same patient was verified by western blot. E,F, Sp2 knockdown was significantly down‐regulated GRP78 in HCC cells, especially in the ATF6 pathway. *P < .05 **P < .01

Previous studies had investigated the changes of super‐enhancers in HCC cell lines before and after ERS by Chromatin immunoprecipitation and high‐throughput sequencing (ChIP‐seq). The target genes of specific super‐enhancers were analyzed in combination with the sequencing results of HCC cells transcriptome. The results showed that some genes were differentially expressed under ERS and might be regulated by super‐enhancers. Transcription factor Sp2 might play a regulation role by combining with these ERS specific super‐enhancers. In order to further clarify the function of Sp2 in HCC and its relationship with ERS, IHC was used to detect the expression of ERS marker protein GRP78 in 95 cases of primary HCC and ANT liver tissues (Figure 5A), while WB method was used to detect 4 pairs of fresh HCC/ANT (Figure 5D). We found that the expression of Sp2 in HCC was similar to that of GRP78. Compared with the corresponding paracancerous tissue specimens, Grp78 was significantly overexpressed in HCC tissue specimens, and Sp2 had a higher expression trend in the high ERS HCC tissues (Figure 5B,C). In vitro experimental results indicated that GRP78 has been dramatically down‐regulated in the Sp2 knockout HCC cells, especially in ATF6. But the PERK and IRE1 were not down‐regulated (Figure 5E,F). Based on this, it could be concluded that Sp2 may greatly influence both the occurrence and development of HCC by enhancing the level of ERS.

### 3.5 Sp2 acts on ERS by regulating positive feedback of TRIB3

**Figure 6:**
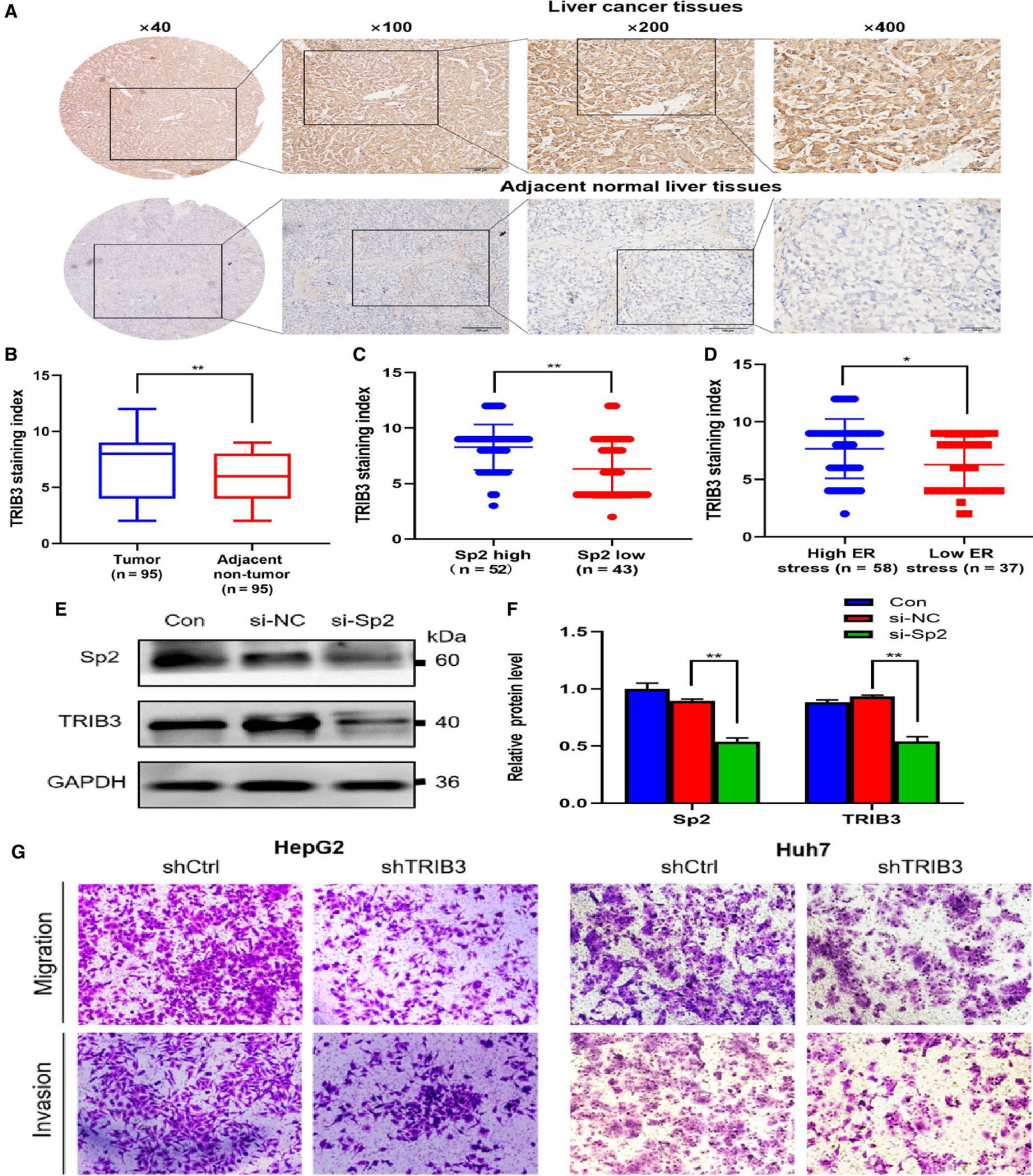
TRIB3 overexpression in HCC and may be targeted by Sp2. A,B, TRIB3 expression in HCC tissue microarrays (A). Compared with the normal samples in the surrounding area, the expression of TRIB3 had a much higher staining index (SI). (B). C, TRIB3 had a higher expression trend in the Sp2‐high expression HCC tissues. D, TRIB3 had a higher expression trend in the high ERS HCC tissues. E,F, Sp2 knockdown was significantly down‐regulated TRIB3 in HCC cells. G, Transwell assay suggested that cell migration and invasion were inhibited both in HepG2 and Huh7 cell lines after knock‐down of TRIB3 (Figure 6G).*P < .05 **P < .01

TRIB3 gene is differentially expressed under ERS and non‐stress conditions. For purpose of further explore the molecular mechanism of Sp2 action, we used IHC to detect the expression of TRIB3 in 95 cases of HCC and ANT tissues (Figure 6A,B). The analysis showed TRIB3 had a higher express trend in the high Sp2 expression (Figure 6C) and ERS HCC tissues (Figure 6D). Use western blot to evaluate the expression of TRIB3 gene after Sp2 knockout. It was found that Sp2 silencing could down‐regulate the TRIB3 expression in HCC cells (Figure 6E,F). Cell migration and invasion assays were performed after knock‐out of TRIB3 in HepG2 and Huh7 cell lines. Transwell assay suggested that cell migration and invasion were inhibited both in the two cell lines after knock‐down of TRIB3 (Figure 6G). Therefore, we speculated that TRIB3 was regulated by ESR, Sp2 may act on TRIB3 protein and enhance its expression, and further heighten ERS by positive feedback, thus playing a major part in proliferation, invasion, and metastasis of HCC.

## 4 DISCUSSION

This study confirmed the role of Sp2 in the carcinogenesis and development of HCC and explored its possible mechanism. The results of WB and IHC study confirmed that SP2 expression was conspicuously up‐regulated in tumor tissues of HCC, which was strongly linked with the progression and poor prognosis of HCC patients. In vitro studies show that Sp2 knockout can significantly inhibit the proliferation and colony formation of HCC cells, induce apoptosis, inhibit migration and invasion. Further experiments show that Sp2 silencing can down‐regulate the expression of TRIB3 and the level of ERS. These findings emphasize that Sp2, as a transcription factor, may combine with enhancers to act on TRIB3 protein, further regulate endoplasmic reticulum stress of HCC, and promote the occurrence and progression of HCC.

Sp2 is one of the transcription factors of the SP family. Previous results show that SP may play a key role in the emergence, evolution, and metastasis of tumors.^13^ Most research mainly explores the effects of Sp1 in human cancer. Sp1 is a crucial transcription factor that regulates the growth of cancer cells. Its abnormal expression regulates the proliferation and metastasis of tumors through complex mechanisms.^14^, ^15^ Researches showed Sp1 is highly expressed in gastric cancer,^16^ HCC,^17^ ovarian cancer,^18^, ^19^ pancreatic cancer 20 and other tumor cells. At the same time, it shows that Sp1 may further promote tumorigenesis and development by regulating VEGF expression.^16^, ^19^ However, there are few reports on the relationship between Sp2 and cancer. Our results have found that the high expression of SP2 is positively related to a series of factors in HCC, which include poor prognosis, clinical stage, lymph node metastasis, and tumor size. The TCGA data further demonstrates the above results. In addition, Zhao and his collaborators 12 have also carried out research on this, and the results confirm that there is an up‐regulation of SP2 expression in human gastric cancer tissue, miR‐638 may induce G1 phase cell cycle arrest by down‐regulating the expression of Sp2. Phan D explored the relationship between Sp2 and prostate cancer.^10^ They found that for prostate cancer suppressor gene CEACAM1, the main reason for its down‐regulation is the strong expression of SP2. These findings are consistent with our findings. Therefore, we believe that Sp2 may be a potential biomarker of high invasiveness and poor prognosis in HCC. It is further found that the down‐regulation of Sp2 not only decreases the proliferation rate of HCC cells but also significantly inhibits the invasion and migration ability of cells, as well as promoted the apoptosis of tumor cells.

Previous studies have found that differentially expressed genes under endoplasmic reticulum stress may be regulated, and TRIB3 protein is among them. TRIB3 belongs to a member of the mammalian pseudokinase tribbles family, which plays a crucial part in unfolded protein response (UPR).^21^ It is closely related to various biological processes as an important stress‐related gene.^22^ As early as 2005, Ohoka has reported that TRIB3 is a novel endoplasmic reticulum stress‐inducing gene.^9^ During the growth of tumors, a series of endoplasmic reticulum stress inducements, such as glucose deficiency and hypoxia, thus up‐regulating the TRIB3 expression. And TRIB3 could further regulate the downstream biological process of endoplasmic reticulum stress, alleviate cell stress and promote cell survival. We speculate that Sp2 may play a role in promoting cancer by regulating the expression of TRIB3 protein. The experiment discovered that the expression of TRIB3 and GRP78 in Sp2 silenced hepatoma cells decreased significantly. IHC and correlation analysis confirmed that the expression trend of TRIB3 and GRP78 is consistent with Sp2 in HCC tissues; TRIB3 levels were positively correlated with Sp2, which tally with our speculation.

Many kinds of research indicate that TRIB3 promotes the emergence and gradual growth of various types of cancers, such as gastric cancer,^23^ renal cancer,^24^ lung cancer,^25^ oral cancer,^26^ and is closely related to poor prognosis. Dong et al^23^ reported that TRIB3 is overexpressing in human gastric cancer, which was associated with the tumor angiogenesis and poor prognosis. Hong et al^24^ found that TRIB3 plays a vital role in the biological processes of migration and invasion of renal cancer cells by regulating the MAPK pathway. And the high expression of TRIB3 is associated with tumor stage and poor prognosis. Zhou et al^25^ reported that knocked out TRIB3 in invasive lung cancer cell lines could inhibit malignant behavior in vitro significantly, such as cell invasion and proliferation, and inhibit the metastasis and growth of tumors in vivo. These findings are consistent with our research, suggesting that TRIB3 seems a key player in promoting the proliferation, migration and invasion of cancer cells. However, some studies have found inconsistent findings. Qu et al^27^ point out that TRIB3 has a higher expression in human endometrial cancer than in benign endometrial tissue and normal tissues. Still, biological experiments showed TRIB3 has a tumor suppressing effect in endometrial cancer, which can promote tumor cell apoptosis and reduce tumor cell proliferation and migration. A study by Wennemers reported that although most of the data make clear elevated TRIB3 mRNA level was associated with poor prognosis, but it was associated with a good prognosis in human breast cancer patients.^28^ The specific causes and mechanisms of these differences remain unclear, which may be related to the mechanism of TRIB3 participating in endoplasmic reticulum stress‐induced tumor cell apoptosis through CHOP/ATF4 pathway at the same time.^29^, ^30^ In addition, we found that knockout of SP2 significantly reduced the protein level of ATF6 but not PERK and IRE1. Under endoplasmic reticulum stress, three pathways of unfolded protein response were activated by three transmembrane proteins that include IRE1, PERK, and ATF6.^6^ The role of ERS mediated by PERK and IRE1 in the pro‐survival or pro‐apoptotic regulatory mechanism is still controversial, which is believed to be the result of multiple factors including micro‐environment, cell types, and stress conditions.^31^, ^32^ Compared with PERK and IRE1, ATF6 is considered to more involved in cell survival mechanism under endoplasmic reticulum stress.^33^ Moreover Numerous studies suggest that the activated ATF6 signaling pathway is correlated with a low overall survival, tumor growth, relapse, and metastasis as well as resistance to chemotherapy in different cancer types.^34^ In combination with the experimental results, it can be speculated that the feedback of SP2 and TRIB3 to endoplasmic reticulum stress after their binding is embodied in their regulation on ATF6 signaling pathway which promotes the survival of cancer cells via cellular protection mechanism. In this study, the role of Sp2 in promoting cancer may be related to the feedback effect of TRIB3 in ERS. The transcription factor Sp2 may bind to the ERS specific super‐enhancer, act on TRIB3 protein and enhance its expression, and further heighten ERS by positive feedback. ERS signaling pathway reduces endoplasmic reticulum pressure by enhancing protein folding ability and the degradation of misfolded protein, ensuring the continued survival of tumor cells, thereby playing a vital part in promoting HCC’s occurrence and evolution.

In conclusion, this study provides the first evidence that Sp2 plays a vital part in the emergence and evolution of HCC. The function may be related to the regulation of TRIB3 protein and ERS. Although the specific mechanism is not yet clear, and also need to further follow‐up experiments to clarify. However, the current study may offer new insights into HCC's pathogenesis and therapeutic targets. As mentioned above, the role of TRIB3 in malignant tumors is still controversial, and further work is needed to understand its role in ERS response pathway fully. More studies are required to elucidate the effects of Sp2 and TRIB3 expression on the occurrence, development and prognosis of HCC.

## CONFLICT OF INTEREST

The authors declare no conflict of interest.

## AUTHOR CONTRIBUTIONS

Guoping Sun initiated and designed the study, Yue Zhu and Jie Cui carried out relevant experiments, Wei Hua collected samples and provided clinical information, Jiatao Liu helped perform the analysis with constructive discussions, Yue Zhu wrote the manuscript. Wei Wei supervised the study. All authors have read and approve the final manuscript.

## Data Availability

The datasets during and/or analyzed during the current study available from the corresponding author on reasonable request.
